# Relation between antioxidant status and postpartum anestrous condition in Murrah buffalo

**DOI:** 10.14202/vetworld.2015.1163-1166

**Published:** 2015-10-09

**Authors:** Mayukh Ghosh, Meenakshi Gupta, Rajesh Kumar, Sunil Kumar, A. K. Balhara, Inderjeet Singh

**Affiliations:** 1 Department of Veterinary Physiology and Biochemistry, Lala Lajpat Rai University of Veterinary and Animal Sciences, Hisar - 125 004, Haryana, India; 2 Department of Veterinary Physiology, Veterinary College, Pookode, Lakkidi - 673 576, Kerala, India; 3 Division of Animal Reproduction, Indian Veterinary Research Institute, Izatnagar, Uttar Pradesh, India; 4 Division of Animal Physiology and Reproduction, Central Institute for Research on Buffalo, Hisar - 125 001, Haryana, India

**Keywords:** antioxidants, postpartum anestrous, stress

## Abstract

**Aim:**

Objective of the present study was to investigate the relation between antioxidant status and postpartum anestrous (PPA) condition in Murrah buffalo.

**Materials and Methods:**

Jugular blood samples were collected from two different groups of Murrah buffaloes each group consisting of 20 animals. Group I was of PPA and Group II were of cyclic buffaloes. The animals selected were examined for confirmation for cyclic and acyclic condition (>120 days) after calving by routine transrectal ultrasonography. Heard record was also used for cross confirmation.

**Results:**

The analysis of antioxidants in plasma and hemolysates revealed that the levels of vitamin E, β-carotene and reduced glutathione in plasma and superoxide dismutase (SOD) in hemolysate were significantly higher in cyclic animals than PPA animals. The levels of vitamin C, SOD and glutathione peroxidase in plasma did not show any significant difference among the two groups studied. The low antioxidant level in affected animals may predispose them toward PPA condition.

**Conclusion:**

Stress imposed by pregnancy and lactation affected the reproductive performance in PPA animals which might be inherently more susceptible to these stressors than those who were normal cyclic as all the animals were maintained under similar feeding and management practices.

## Introduction

As the second largest source of milk in the world and about 56.5% of total milk production in India, Buffalo bear premier importance in dairy industry along with its significant contribution in foreign exchange earnings by export of meat [1]. The low reproduction efficiency in buffalo is the major constraint in obtaining maximum production potential set a perfect platform for the current research input. Among the various factors reducing its reproductive efficiency, postpartum anestrous (PPA) is a vital anomaly [2,3]. Many factors contributing individually or in concert were recognized to be responsible for postpartum infertility and anestrous making it a complex phenomenon. In ruminants, these factors include nutrients, mineral deficiencies [4], season [5], suckling [6], parity [7], infection [8,9], and dystocia[10], etc.

Stress responses in heat, pregnancy and milk production lead to formation of reactive oxygen and nitrogen species (ROS and RNS). These ROS and RNS include hydroxyl radicals, superoxide ion, hydrogen peroxide, nitric oxide radicals and are involved in free radical chain reaction affecting lipid peroxidation, apoptosis, and fertility [11]. The biological consequences of these ROS and RNS mediated free radical chain reaction leads to infertility by affecting folliculogenesis, steroidogenesis, and preimplantation of an embryo which are sensitive to free radical damage [12]. Antioxidant defense system mitigates the free radical damage by disposing these ROS and blocking the free radical chain reaction to keep the animals healthy. The enzymatic components of this defense system mainly comprises of superoxide dismutase (SOD), catalase, and glutathione peroxidase (GPX) whereas the non-enzymatic counterpart includes reduced glutathione (R-GSH), vitamin C, vitamin E, β- carotene, and different macro and micro elements.

So, hypothesizing that the pro-oxidant and antioxidant balance underlining the animal reproductive efficiency; the present study was designed to explore the relation between antioxidant status and PPA condition in Murrah buffalo.

## Materials and Methods

### Ethical approval

The experiments on animals including all procedures of this study were approved by Institutional Animal Ethics Committee.

### Experimental animals

The present investigation was carried out at the animal farms of Central Institute for Research on Buffaloes, Hisar; and Lala Lajpat Rai University of Veterinary and Animal Science, Hisar. Twenty PPA and twenty normal cyclic Murrah buffaloes were selected on the basis of their reproductive history obtained from farm records. According to the herd records, the buffaloes that had shown anestrous for more than 120 days were selected in postpartum group (PPA) and animal coming in estrous before 65 days of postpartum for more than three consecutive lactations including present lactation were selected in normal cyclic group for conducting the study. The animals selected in the current lactation were having average postpartum anestrous period of 191.47±13.37 days and those in normal cyclic had 60.64±5.38 days. Current status of reproductive organs of all animals in the study was also examined and verified by per rectal examination and ultrasonography. The animals were maintained as per the standard feeding and management practices followed at the farms and were fed for body maintenance and according to the level of milk production, so that the green and dry fodder were appropriately supplemented with concentrate mixture containing mineral mixture. Buffaloes were loose housed in open and closed paddock.

### Collection and transportation of blood samples

Approximately,10 ml of jugular blood sample was collected from each experimental animal in 15 ml sterile polypropylene centrifuge tube containing ethylenediaminetetraacetic acid as anticoagulant in the month of November and December. Plasma was separated in refrigerated centrifuge at 3000 rpm for 15 min and stored in aliquots at −20°C until analysis of vitamins and antioxidants. Following separation of plasma from blood samples by centrifugation, the white blood cells layer was separated, and the remaining erythrocytes were washed thrice with a cold normal saline solution. Then distilled water was added to erythrocyte pellet slowly and with constant stirring up to 1:1 dilution to prepare hemolysate. It was stored at −20°C for estimation of SOD. All chemicals used in this study were procured from Sigma-Aldrich chemicals, USA.

Nitric oxide scavenging activity of plasma was measured by the method of Sreejayan and Rao [13]. R-GSH assay was performed by the method of Beutler [14]. Plasma GPX-3 was estimated by kit provided by Cayman Chemical Company, U.S.A. The activity of SOD in red blood cells (RBC) hemolysate and plasma was measured by the method of Madesh and Balasubramaniam [15]. Vitamin E was estimated in plasma by method of Kayden *et al*. [16]. Estimation of vitamin C in plasma was done by 2,4-dinitrophenylhydrazine. Plasma β- carotene was estimated by the method of Baker *et al*. [17] and plasma protein by commercially available kit (Total Protein Kit, Coral Clinical System, India).

### Statistical analysis

All the data were expressed as mean±standard error values. Statistical analyses were carried out using GraphPad Prism v6.0 (GraphPad Software, San Diego, CA, USA) software implementation of Student’s *t-*test.

## Results

### Vitamins

Plasma vitamin E, β-carotene, and vitamin-C levels were compared between PPA and normal cyclic animals. Vitamin E and β-carotene levels were observed to be significantly (p<0.05) higher in normal cyclic animals in comparison to PPA animals whereas vitamin-C level did not differed significantly between the two groups (Table-1).

**Table-1:** Comparison of mean (±SE) values of plasma vitamins between PPA and normal cyclic Murrah buffaloes.

Parameters	PPA	Normal	p value
Vitamin C (mg/dl)	0.697±0.137	1.193±0.262	0.115
Vitamin E (µg/dl)	195.299±25.562	335.037±52.656*	0.029
β carotene (µg/ml)	1.970±0.303	3.494±0.563*	0.030

* Significant at p<0.05, PPA=Postpartum anestrous, SE=Standard error

### Antioxidants

The mean plasma nitric oxide scavenging activity, plasma R-GSH, plasma GPX-3, plasma SOD and SOD in RBC between PPA and normal cyclic animals along with their standard error has been depicted in Table-2. Plasma R-GSH concentration was found to be significantly higher (p<0.05) higher in normal cyclic animals than PPA animals. In terms of enzymatic antioxidants, SOD activity in RBC was found to be significantly (p<0.01) higher in normal cyclic animals but the plasma SOD activity and GPX-3 activity did not differed significantly (p<0.05) between the two groups of animals while the difference in nitric oxide scavenging activity was also found to be non-significant (p<0.05) between the two groups.

**Table-2:** Comparison of mean (±SE) values of plasma protein, plasma nitrous oxide scavenging activity, plasma reduced GSH, plasma GPX-3, plasma SOD and SOD in RBC between PPA and normal cyclic animals.

Parameter	PPA	Normal	p value
Protein (g/dl)	7.544±0.117	8.034±0.121**	0.007
Nitrous oxide scavenging activity (%)	33.421±2.525	36.648±2.149	0.339
Reduced GSH (µmol)	208.908±3.954	225.338±4.206**	0.008
GPX3 (IU/g protein)	1.939±0.128	1.779±0.125	0.698
SOD plasma (units/ml)	1.160±0.203	0.968±0.160	0.465
SOD RBC (units/g Hb)	132.18±19.48	224.679±24.800**	0.009

** Significant at p<0.01, GSH=Glutathione, GPX3=Glutathione peroxidase, PPA=Postpartum anestrous, SOD=Superoxide dismutase, RBC=Red blood cells, SE=Standard error

### Plasma protein

The total plasma protein concentration was observed to be significantly (p<0.01) higher in normal cyclic animals (Table-2).

## Discussion

In the present study, vitamin E was significantly (p<0.05) higher in normal cyclic animals than PPA group which is in corroborate the finding of Kahlon and Singh [18], Surapaneni and Vishnu [19]. This indicates that animal showing PPA is under oxidative stress resulting in decreased fertility. Similarly, analysis of data revealed significantly (p<0.05) lower level of β-carotene in PPA than normal cyclic animals. This may be due to that β-carotene is consumed during scavenging of free radicals. Similar reports such as Weiss [20] Jukola *et al*. [21] and Derar *et al*. [22] in cattle are also in accordance with the current findings. The non-significant variation in vitamin C level in both groups is in agreement with Serpek *et al*. [23].

The higher level of plasma GSH concentration in normal cyclic animals is also corroborate with the experiments of Ahmed *et al*.[24], Hanafi *et al*. [25] and Ahmed *et al*. [26], who reported similar observation in anestrous or non-cyclic animals. Similarly, Ahmed *et al*. [26] reported significant decrease in erythrocytic R-GSH in non-cyclic heifer buffalo than normal cyclic. Surapaneni and Vishnu [19] observed significantly lower level of erythrocytic GSH in polycystic ovary syndrome compared to normal cycler female in human. This indicates that PPA animals were in oxidative stress, and decrease in the levels of these non-enzymatic antioxidants values were probably due to increased turnover for prevention of oxidative damages because the role of the antioxidants is to prevent the generation of free radicals and to nullify the effect of free radicals [27].

SOD converts ROS generated by cell to hydrogen peroxide by spontaneous dismutation [28]. In our study, significantly higher level (p<0.01) of erythrocytic SOD in normal cyclic animals than PPA group animals was observed. Whereas plasma SOD did not differ significantly between normal cyclic and PPA group animals. The results are in accordance with related reports in buffaloes suffering from heat stress [24,26,29]. Whereas Kahlon and Singh [18] in anestrous buffaloes and Surapaneni and Vishnu [19] in polycystic ovary syndrome animals reported opposite effect. This indicates PPA animals were in oxidative stress, and decrease in the levels of erythrocytes SOD values were probably due to increased turnover for prevention of oxidative damages. Whereas plasma SOD showed non-significant variation between both the groups as plasma SOD indicates short-term stress condition.

The current study revealed that plasma GPX-3 level was not significantly different in both group. Similar reports for dairy cow are also documented in related experiments [30].

Significantly higher protein in normal cyclic animals than PPA buffaloes has been reported by Amanullah *et al*. [31] and Kumar *et al*. [32]. The present study also confirms these previous findings. So, our results indicate that amelioration of production stress is essential to maintain reproductive efficiency in female animals [33].

## Conclusion

The vitamin E, β-carotene, reduced glutathione and protein investigated in plasma of buffaloes suffering from PPA were significantly (p<0.05) lower than normal cyclic buffaloes. The SOD level in hemolysate was also significantly (p<0.01) lower in PPA group animal than normal cyclic animals. Whereas plasma vitamin C level, SOD, GPX-3, and nitric oxide scavenging activity (%) were non-significantly (p<0.05) different in both groups. This indicates buffaloes under PPA condition are suffering from stress which may be because of production or reproduction. It also indicates that these animals are more susceptible to stress and apart from normal feeding additional supplementation of antioxidant vitamins can be beneficial to them at time of stress to optimize the performance in buffaloes.

## Authors’ Contributions

RK, MG, and AKB have designed the study and planned the research experiment. RK, MG, and SK performed the research experiments. IS, AKB, and MG supervised the research and performed manuscript preparation. All the authors read and approved the final manuscript.
